# The role of CaMKII regulation of phospholamban activity in heart disease

**DOI:** 10.3389/fphar.2014.00005

**Published:** 2014-01-27

**Authors:** Alicia Mattiazzi, Evangelia G. Kranias

**Affiliations:** ^1^Facultad de Medicina, Centro de Investigaciones Cardiovasculares, Conicet La Plata-Universidad Nacional de La PlataLa Plata, Argentina; ^2^Department of Pharmacology and Cell Biophysics, College of Medicine, University of CincinnatiCincinnati, OH, USA

**Keywords:** myocardium, CaMKII, PLN regulation, acidosis, ischemia/reperfusion injury, heart failure

## Abstract

Phospholamban (PLN) is a phosphoprotein in cardiac sarcoplasmic reticulum (SR) that is a reversible regulator of the Ca^2^^+^-ATPase (SERCA2a) activity and cardiac contractility. Dephosphorylated PLN inhibits SERCA2a and PLN phosphorylation, at either Ser^16^ by PKA or Thr^17^ by Ca^2^^+^-calmodulin-dependent protein kinase (CaMKII), reverses this inhibition. Through this mechanism, PLN is a key modulator of SR Ca^2^^+^ uptake, Ca^2^^+^ load, contractility, and relaxation. PLN phosphorylation is also the main determinant of β1-adrenergic responses in the heart. Although phosphorylation of Thr^17^ by CaMKII contributes to this effect, its role is subordinate to the PKA-dependent increase in cytosolic Ca^2^^+^, necessary to activate CaMKII. Furthermore, the effects of PLN and its phosphorylation on cardiac function are subject to additional regulation by its interacting partners, the anti-apoptotic HAX-1 protein and Gm or the anchoring unit of protein phosphatase 1. Regulation of PLN activity by this multimeric complex becomes even more important in pathological conditions, characterized by aberrant Ca^2^^+^-cycling. In this scenario, CaMKII-dependent PLN phosphorylation has been associated with protective effects in both acidosis and ischemia/reperfusion. However, the beneficial effects of increasing SR Ca^2^^+^ uptake through PLN phosphorylation may be lost or even become deleterious, when these occur in association with alterations in SR Ca^2^^+^ leak. Moreover, a major characteristic in human and experimental heart failure (HF) is depressed SR Ca^2^^+^ uptake, associated with decreased SERCA2a levels and dephosphorylation of PLN, leading to decreased SR Ca^2^^+^ load and impaired contractility. Thus, the strategy of altering SERCA2a and/or PLN levels or activity to restore perturbed SR Ca^2^^+^ uptake is a potential therapeutic tool for HF treatment. We will review here the role of CaMKII-dependent phosphorylation of PLN at Thr^17^ on cardiac function under physiological and pathological conditions.

## INTRODUCTION

A major characteristic of human and experimental heart failure (HF) is altered Ca^2^^+^ cycling, associated with decreased contractility, which partially reflects the impaired function of the sarcoplasmic reticulum (SR) membrane. During a normal excitation-contraction-coupling cycle (ECC), Ca^2^^+^ enters the cell through the L-type Ca^2^^+^ channels leading to activation of the ryanodine receptors (RyR2) in the SR and release of Ca^2^^+^ from this membrane system. This Ca^2^^+^-induced-Ca^2^^+^-release mechanism (Fabiato and Fabiato, 1977) underlies a fine-tuned synchronization of Ca^2^^+^ cycling in the heart, coordinating contraction and relaxation. Relaxation is mediated mainly by the activity of the SR Ca^2^^+^-ATPase (SERCA2a) and to a lesser extent by the Na^+^/Ca^2^^+^ exchanger (NCX). Thus, the SR is the major regulator of Ca^2^^+^-handling during the cardiac excitation-contraction-relaxation cycle (Bers, 2001).

The activity of SERCA2a is under the reversible control of phospholamban (PLN), an SR associated protein (Tada et al., 1975). PLN is a 52 amino acid phosphoprotein, which, in the dephosphorylated state, inhibits the apparent Ca^2^^+^-affinity of SERCA2a (James et al., 1989; Kim et al., 1990). PLN can be phosphorylated at three distinct sites in vitro: Ser^16^ by cyclic AMP (cAMP)- and cGMP-dependent protein kinases, Thr^17^ by Ca^2^^+^-calmodulin-dependent protein kinase II (CaMKII), and Ser^16^ by protein kinase C (Movsesian et al., 1984; Simmerman et al., 1986; Huggins et al., 1989). Phosphorylation of these sites *in vitro* relieves the inhibition of PLN on SERCA2a and increases SR Ca^2^^+^ uptake. Whereas Ser^10^ phosphorylation by PKC does not occur in intact hearts (Edes and Kranias, 1990), cGMP phosphorylation of PLN has been described in isolated myocytes (Bartel et al., 1995). However, the physiological significance of this pathway is still unclear. In contrast, phosphorylation of Ser^16^ and Thr^17^ by PKA and CaMKII has been shown to be a key mediator of the positive inotropic and relaxant effects of ß1-adrenergic stimulation in the heart. The increase in SERCA2a activity and Ca^2^^+^ uptake rate elicited by the phosphorylation of these sites, leads to an increase in the velocity of relaxation, SR Ca^2^^+^ load and SR Ca^2^^+^ release which, in association with L-type Ca^2^^+^ channel and RyR2 phosphorylation, mediate the enhanced contractility produced by ß1-stimulation (Lindemann et al., 1983; Lindemann and Watanabe, 1985; Vittone et al., 1990; Napolitano et al., 1992; Mundiña-Weilenmann et al., 1996; Kuschel et al., 1999). Dephosphorylation of PLN, occurring by a SR-associated type 1 phosphatase (PP1; MacDougall et al., 1991), reverses the activation of SERCA2a and the stimulatory effects of β1-agonists. This article will discuss the role of Thr^17^ phosphorylation of PLN and address its significance under physiological and pathological processes.

## THE PHOSPHOLAMBAN REGULATOME

Phospholamban was first described as a cAMP-dependent protein kinase substrate in the early 1970s. The phosphorylated amino acid was shown to be Ser^16^ and phosphorylation enhanced SERCA2a activity and Ca^2^^+^-uptake (Kirchberger et al., 1972). Subsequently, PLN was shown to be also phosphorylated by a SR-associated Ca^2^^+^-CaM-kinase (CaMKII) at Thr^17^ and this phosphorylation occurred independently of its PKA-phosphorylation (Bilezikjian et al., 1981; Davis et al., 1990). Phosphorylation by CaMKII also enhances SR Ca^2^^+^-transport through an increase in the apparent affinity of the SERCA2a for Ca^2^^+^ (K_Ca_). Thus, it was initially proposed that phosphorylated PLN acts as a stimulator of cardiac SERCA2a activity. However, in the late 1980s and early 1990s, there were two significant breakthroughs: (a) *in vitro* studies of reconstituted SR membrane systems (James et al., 1989; Kim et al., 1990); and (b) *in vivo* studies in mouse models with ablation or overexpression of PLN (Luo et al., 1994, 1996; Kadambi et al., 1996), which demonstrated that dephosphorylated PLN is actually an inhibitor of SERCA2 and phosphorylation relieves this inhibition, giving the appearance of phosphorylation-induced stimulation. These findings, together with the characterization and identification of a cardiac SR–associated protein phosphatase that can dephosphorylate PLN (Kranias, 1985), has led to our current understanding of PLN as a reversible inhibitor of cardiac SR Ca^2^^+^-ATPase activity.

Furthermore, recent studies showed that the activity of PLN can itself be regulated by the HS-1 associated protein X-1 (HAX-1), which is ubiquitously expressed in mitochondria and SR. HAX-1 physically interacts with PLN and the binding region of PLN includes amino acids 16–22 with both Ser^16^ and Thr^17^ phosphorylation sites. Interestingly, phosphorylation of PLN diminishes its binding to HAX-1, indicating that this interaction may be physiologically relevant in the heart (Vafiadaki et al., 2007). Indeed, HAX-1 has been found to increase PLN inhibition of SR Ca^2^^+^ cycling and cardiac contractility *in vivo,* whereas β1-adrenergic stimulation relieves this inhibition (Zhao et al., 2009; Lam et al., 2013).

**FIGURE 1:**
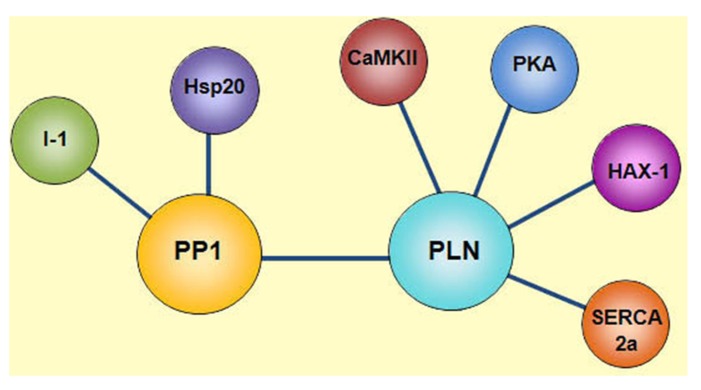
**Phospholamban regulatome.** Scheme of the multimeric protein complex constituted by SERCA2a, PLN, HAX-1, PKA, CAMKII, PP1, Inhibitor-1 (I-1), and Hsp20, which reversibly regulates SR Ca^2^^+^ transport in the cell. SERCA2a activity is regulated by its reversible inhibitor PLN and the histidine rich Ca^2^^+^-binding protein (HRC). Phosphorylation of PLN is mediated by cAMP-dependent protein kinase (PKA) at Ser^16^ site and Ca^2^^+^-calmodulin-dependent protein kinase (CaMKII) at Thr^17^ site. Dephosphorylation of these sites occurs by protein phosphatase 1 (PP1). The activity of PP1 is regulated by inhibitor-1 (I-1) and Hsp20.

Besides HAX-1, other regulatory proteins such as PKA, CAMKII and PP1 are also associated with PLN, achieving an efficient and compartmentalized complex that regulates SR Ca^2^^+^-cycling and cardiac function. PP1 is a negative regulator of PLN activity through its dephosphorylation and increased inhibition of SERCA2a. Interestingly, the type 1 enzyme is modulated by its endogenous inhibitors, Inhibitor-1 (I-1) and Inhibitor-2 (I-2). Inhibitor-1 gets activated upon its PKA phosphorylation at Thr-35 resulting in potent inhibition of PP1 activity and amplification of the β1-adrenergic receptor stimulatory effects (Iyer et al., 1988; Neumann et al., 1991; Gupta et al., 1996). More recently, the small heat shock protein 20 (Hsp20) was also identified as a novel interacting partner of PP1 and inhibitor of its enzymatic activity, resulting in diminished PLN inhibition and enhanced cardiac function (Qian et al., 2011). Thus, there is a multimeric functionally coupled signaling complex, which reversibly regulates SR Ca^2^^+^ cycling in the cell, composed of SERCA, PLN, HAX-1, PKA, CAMKII, PP1, I-1, and Hsp20 (**Figure 1**).

## PHOSPHORYLATION OF PLN BY CaMKII UNDER PHYSIOLOGICAL CONDITIONS

### ß1-ADRENERGIC STIMULATION

**FIGURE 2:**
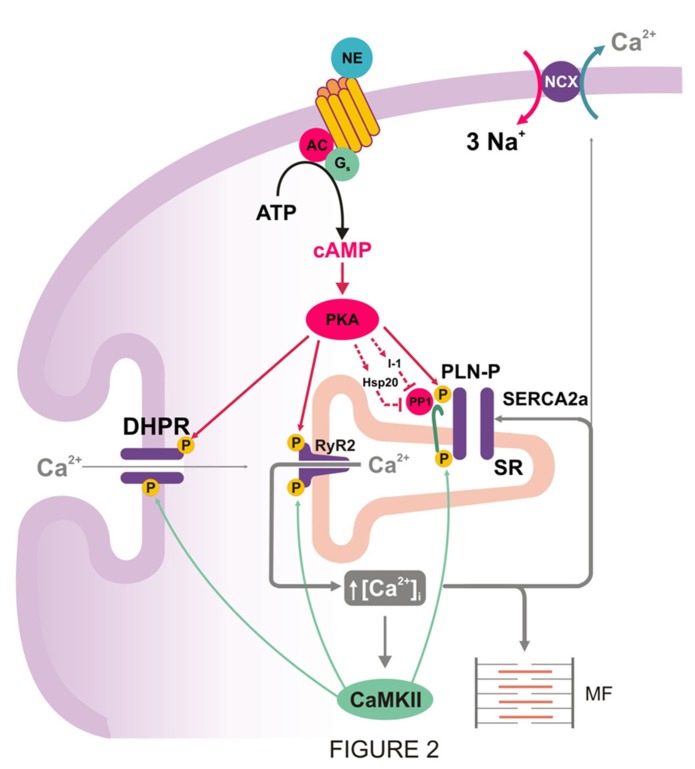
**PKA mediated increase in cytosolic Ca^**2**^^**+**^ and inhibition of PP1: two prerequisites for CaMKII-dependent phosphorylation of PLN during β1-adrenergic stimulation.** PKA-dependent phosphorylation of Ca^2^^+^ handling proteins, particularly L-type Ca^2^^+^ channel and PLN, produces an increase in cytosolic Ca^2^^+^ that is necessary to activate CaMKII and produce CaMKII-dependent phosphorylation. PKA also increases inhibitor-1 and Hsp20 phosphorylation, amplifying the stimulatory effects of β1–adrenergic stimulation on SR Ca^2^^+^-transport, relaxation, and contractility.

It is well established that stimulation by β1-agonists at the cell membrane, initiates a signal-transduction pathway that involves the Gs proteins to stimulate cAMP formation by adenylate cyclase, followed by PKA activation (**Figure 2**). PKA then phosphorylates several proteins in the cardiac myocytes to induce positive chronotropic, inotropic, and relaxant effects, the so-called “fight or flight response,” which is considered the most effective mechanism to acutely increase cardiac output. The underlying phosphoproteins include PLN and RyR2 at the SR level, the L-type Ca^2^^+^ channel and phospholemman, at the sarcolemma level, and troponin I (TnI), C protein, and myosin light chain, at the level of the myofibrils (Bers, 2001).

The role of PLN phosphorylation vs. the phosphorylation of other proteins, which are also involved in ECC, was demonstrated by the generation and characterization of gene knockout and transgenic models with ablation, reduction or overexpression of PLN in the heart. Ablation of PLN was associated with enhanced affinity of SERCA2a for Ca^2^^+^, contractility and relaxation (Luo et al., 1994, 1996). In contrast, overexpression of PLN decreased Ca^2^^+^affinity of SERCA2a and diminished SR Ca^2^^+^-load and cardiac contractility (Kadambi et al., 1996). Importantly, studies at the cardiomyocyte, organ and intact animal levels from PLN-deficient mice, indicated a significant attenuation of the inotropic and lusitropic effects of isoproterenol, compared with wild types (Luo et al., 1994; Hoit et al., 1995), and revealed that PLN is a major mediator of the β1-adrenergic response in the mammalian heart. However, as noted above, PLN is not only phosphorylated by PKA, at Ser^16^, but also by CaMKII at Thr^17^, during β1-adrenergic stimulation. These phosphorylations appear to occur independently of each other *in vitro* (Bilezikjian et al., 1981; Davis et al., 1990). However, *in vivo* attempts to phosphorylate PLN by CaMKII indicated that an increase in cAMP levels was a requirement for CaMKII activation (Lindemann et al., 1983; Lindemann and Watanabe, 1985; Vittone et al., 1990; Napolitano et al., 1992; Mundiña-Weilenmann et al., 1996; Kuschel et al., 1999). This may be due to required PKA-phosphorylation of PLN, as well as L-type Ca^2^^+^ channels and RyR2, to enhance cytosolic Ca^2^^+^, necessary to activate CaMKII. Indeed the availability of transgenic models, expressing either wild type PLN (PLN-WT), Ser^16^ → Ala mutant PLN (PLN-S16A) or Thr^17^ → Ala mutant PLN (PLN-T17A) in the cardiac compartment of PLN knockout mice, indicated that the phosphorylation of Ser^16^ of PLN is a prerequisite for the phosphorylation of Thr^17^ (Luo et al., 1998). In addition, these studies showed that Ser^16^ can be phosphorylated independently of Thr^17^ *in vivo* and that phosphorylation of Ser^16^ was sufficient for mediating the maximal cardiac responses to β1-adrenergic stimulation (Chu et al., 2000).

The role of CaMKII-phosphorylation of PLN was also addressed in a model with expression of a CaMKII inhibitory peptide targeted to the longitudinal SR (AIP4-LSR TG; Ji et al., 2006). The results indicated that Thr^17^ PLN-phosphorylation as well as SR Ca^2^^+^-uptake and contractile parameters were decreased. However, the response to isoproterenol remained unaltered. Similarly, transgenic mice with CaMKII inhibition (AC3-I mice), decreased SR Ca^2^^+^-content without changes in the myocyte response to isoproterenol (Zhang et al., 2005). These findings suggested a predominant role of Ser^16^ phosphorylation over that of Thr^17^ in the β1-adrenergic response. Furthermore, experiments in perfused rat hearts using the PKA inhibitor H-89, confirmed that PKA activation is required for β1-adreniceptor mediated phosphorylation of the Thr^17^ site in PLN (Said et al., 2002). It was further demonstrated that, when both PLN phosphorylation sites are present, the CaMKII site contributes to PLN phosphorylation and enhanced mechanical effects only at relatively high levels of β1-adrenergic stimulation, i.e., isoproterenol concentrations ≥10 nM. The lack of contribution of Thr^17^ site to PLN phosphorylation at lower isoproterenol concentrations was attributed to a moderate increase in PKA activity, which would raise intracellular Ca^2^^+^ to a level not sufficient to activate CaMKII and phosphorylate Thr^17^ site (Mundiña-Weilenmann et al., 1996; Said et al., 2002). Taken together, these findings support the notion that CaMKII is a contributor in the stimulatory effects of β1-adrenergic receptor in the heart. However, PKA activation is required to create the necessary conditions for CaMKII activation and Thr^17^ phosphorylation (**Figure 2**). A similar conclusion should hold true for the different Ca^2^^+^ handling proteins which are phosphorylated by both kinases, like L-type Ca^2^^+^ channels or RyR2. Interestingly, sustained β1-adrenergic receptor stimulation enhanced cell contraction and Ca^2^^+^ transients by a mechanism which is largely PKA-independent but sensitive to CaMKII-inhibitors. In these studies, a shift from Ser^16^ to Thr^17^ phosphorylation pathway was observed, underscoring the role of CaMKII during prolonged β1-adrenergic stimulation (Wang et al., 2004). In addition, β1-adrenoceptors activate the guanine nucleotide exchange protein that is directly activated by cAMP (Epac), independently of, and in parallel with, PKA. Indeed, Oestreich et al. (2009) identified RyR2 and PLN as two effector targets of a pathway mediated by Epac-PLCє-PKCє-CaMKII. These authors described an increase in Ca^2^^+^ transient mainly attributed to an increase in RyR2 sensitivity by Ca^2^^+^ influx activation. Although the specific role of Thr^17^ phosphorylation of PLN in these effects was not directly tested, they showed that β-adrenergic stimulation-mediated enhancement of SR Ca^2^^+^ load and myoplasmic Ca^2^^+^ clearance were not significantly altered by PLCє ablation, suggesting a poor role of the pathway described, on SR Ca^2^^+^ uptake (Oestreich et al., 2007). Moreover, other results showed that Epac activation decreases the amplitude of evoked Ca^2^^+^ transient due to Epac-induced SR Ca^2^^+^ leak by CaMKII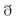-phosphorylation of RyR2 and SR depletion (Pereira et al., 2007, 2013). The different outcomes of the effects of Epac on Ca^2^^+^ transient amplitude may be due to different experimental protocols, since Epac activation produces an initial increase in Ca^2^^+^ transients before reaching a steady state, in which Ca^2^^+^ transients are decreased. Yet, both results are consistent with an increase in RyR2 activation produced by Epac. The more recent study by Pereira et al. (2013) further showed that inhibition of PKA-dependent effects of isoproterenol by H-89 pretreatment blocked the isoproterenol-induced increase of Ca^2^^+^ transient amplitude, speed of relaxation and SR Ca^2^^+^ load. In contrast, isoproterenol still greatly increased SR Ca^2^^+^ spark frequency and decreased Ca^2^^+^ transient amplitude. Both of these effects were similar to the steady state responses produced by Epac activation. These results would imply that: (1) most of the isoproterenol-induced PKA-independent Ca^2^^+^ leak enhancement is mediated by Epac; and (2) the contribution of Epac to isoproterenol–induced SR Ca^2^^+^ reuptake through PLN phosphorylation, is very modest, if any, since no relaxant effects of isoproterenol could be detected after PKA inhibition.

### PHOSPHORYLATION OF Thr^**17**^ OF PLN IN THE ABSENCE OF β1-ADRENERGIC STIMULATION

As indicated above, several studies showed that CaMKII-dependent PLN phosphorylation can only occur in the intact beating heart in the presence of ß1-adrenergic stimulation, while it occurs independently of cAMP-PKA activation *in vitro*. To address this apparent discrepancy, the phosphatase inhibitor okadaic acid was used in the presence of high extracellular Ca^2^^+^. Under phosphatase inhibition, increasing Ca^2^^+^, increased contractility, relaxation and phosphorylation of Thr^17^ of PLN, without significantly changing either cAMP or Ser^16^ phosphorylation (Mundiña-Weilenmann et al., 1996). These findings indicated that Thr^17^ can be phosphorylated independently of Ser^16^ of PLN in the intact heart, in accordance with the *in vitro* studies. Thus, the relative balance of protein kinase (PKA and CaMKII) and phosphatase activities appears to regulate phosphorylation of Thr^17^ and Ser^16^ in PLN.

Stimulation frequency (SF), a fundamental physiological modulator of myocardial performance, is another example in which Thr^17^ phosphorylation of PLN can occur in the absence of prior Ser^16^ phosphorylation (Hagemann et al., 2000; Zhao et al., 2004; Valverde et al., 2005). These findings are in concert with the fact that CaMKII can decode the frequency of Ca^2^^+^ spikes into distinct amounts of kinase activity (De Koninck and Schulman, 1998), and indicate that SF can produce a sustained increase in CaMKII, which leads to the phosphorylation of Thr^17^ in PLN, without the requirement of phosphatase inhibition. Moreover, these results prompted the link between the observed Thr^17^ phosphorylation and the relaxant effect of increasing SF (frequency-dependent acceleration of relaxation or FDAR, Bers, 2001; Hagemann et al., 2000). Indeed, FDAR was inhibited in the presence of CaMKII-inhibitors and in cardiomyocytes expressing the mutant T17A-PLN (Zhao et al., 2004). However, although the involvement of SR and CaMKII in FDAR was supported by several studies (Bassani et al., 1995; DeSantiago et al., 2002; Picht et al., 2007; Wu et al., 2012), a recent report challenged these previous findings by showing that FDAR was still present in CaMKIIδ-KO mice (Neef et al., 2013). These results would suggest that either a CaMKIIδ-independent mechanism or another CaMKII isoform, like CaMKIIγ, is playing a role in FDAR.

The role of Thr^17^ phosphorylation of PLN on FDAR was also questioned on the basis of three main findings: (1) FDAR precedes the phosphorylation of Thr^17^ site of PLN (Valverde et al., 2005; Huke and Bers, 2007); (2) Most studies concur that the main regulatory effect of PLN phosphorylation is to increase the apparent Ca^2^^+^ affinity of SERCA2a (Simmerman and Jones, 1998), while FDAR is associated with an increase in the maximal velocity of SR Ca^2^^+^ uptake (Picht et al., 2007); and (3) FDAR has been also detected in PLNKO mice in one study (DeSantiago et al., 2002), although this finding was not observed in other studies (Bluhm et al., 2000; Wu et al., 2012).

Taken together, the underlying molecular steps that encompass the FDAR process are currently unclear. Although most of the experimental evidence indicates that CaMKII is involved in FDAR, some studies have challenged this possibility and the participation of PLN in FDAR. Thus, it is likely that several rather than a single mechanism, are associated with this phenomenon.

## PHOSPHORYLATION OF PLN BY CaMKII UNDER PATHOLOGICAL CONDITIONS

### ACIDOSIS

#### Mechanical recovery during acidosis

An understanding of how pH changes alter cardiac function is important for a better comprehension of some cardiac pathological situations, which are important in the clinical setting. Myocardial ischemia is particularly relevant along these lines: in human, acidosis can be detected 15 s after the occlusion of the coronary artery and is a major mechanism for the loss of contractility during ischemia (Poole-Wilson, 1989). Substantial changes in intracellular pH may also occur in disorders of different origins which affect cardiac function, like sleep apnea/hypopnea syndrome, diabetic ketoacidosis or in patients on dialysis.

**FIGURE 3:**
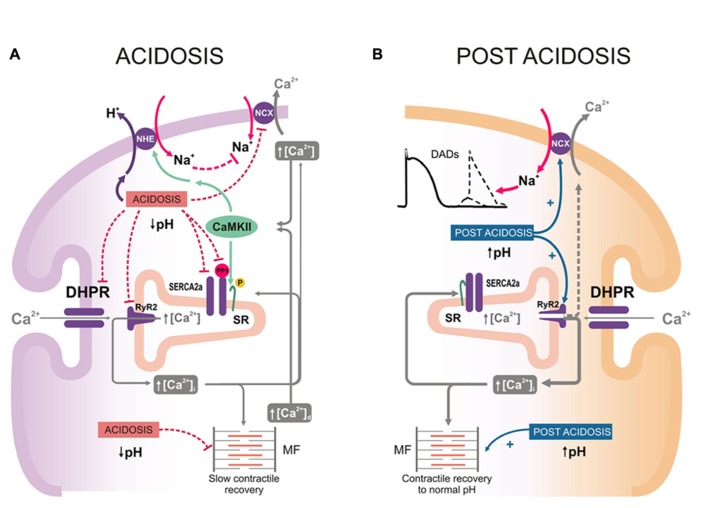
**(A)** Intracellular mechanisms that may contribute to the mechanical recovery during acidosis. Acidosis produces a decrease in myofilament Ca^2^^+^ responsiveness which increases diastolic Ca^2^^+^ ([Ca^2^^+^]_d_). Activation of NHE and direct acidosis inhibition of Na^+^-Ca^2^^+^ exchanger (NCX), would contribute to the increase in cytosolic Ca^2^^+^. Acidosis also inhibits PP1. The simultaneous increase in cytosolic Ca^2^^+^ and inhibition of PP1 activates CaMKII and enhances PLN phosphorylation at Thr^17^ site. As a consequence, there is an increase in SR Ca^2^^+^ uptake, able to offset the direct acidosis-induced inhibition of SERCA2a activity. This would lead to enhanced SR Ca^2^^+^ release and Ca^2^^+^ transients, which counteract the negative effect of acidosis on contractile proteins and supply the substrate for the slow mechanical recovery during acidosis. **(B)**. Putative intracellular mechanisms of post-acidosis induced-arrhythmias. Upon returning to control pH, the inhibitory effects of acidosis are rapidly removed, favoring the increase in Ca^2^^+^ cycling and the contractile recovery towards control levels. However, the relief of RyR2 from the previous constrain produced by acidosis, evokes also an increase in diastolic Ca^2^^+^ leak from the (Ca^2^^+^-loaded) SR. Such release may activate inward currents through the NCX, also relieved from the inhibition evoked by acidosis. The inward Na^+^ current, if large enough, can trigger arrhythmias.

Acidosis produces a rapid decrease in the strength of contraction (Cingolani et al., 1970; Allen and Orchard, 1983), which is largely due to a decrease in myofilament Ca^2^^+^ responsiveness (Fabiato and Fabiato, 1978). This decrease displaces Ca^2^^+^ from troponin C, and would be the main mechanism responsible for the early increase in diastolic Ca^2^^+^ during acidosis. The initial fall in contractility is followed by an increase in the amplitude of intracellular Ca^2^^+^ transients and contractile force (Mattiazzi and Cingolani, 1977a, b; Allen and Orchard, 1983, the mechanism of which is not intuitively obvious, because acidosis inhibits most of the steps of excitation-contraction-coupling (Orchard and Kentish, 1990). Earlier experiments suggested that acidosis-induced activation of Na^+^-H^+^ exchanger (NHE), by increasing cytosolic Na^+^ and then Ca^2^^+^ through the NCX, was sufficient to overcome the inhibitory effect of acidosis on SERCA2a, increasing SR Ca^2^^+^ and intracellular Ca^2^^+^ transients (Harrison et al., 1992). However, inhibition of NHE does not always prevent intracellular Ca^2^^+^ and mechanical recovery (Choi et al., 2000; DeSantiago et al., 2004), indicating that additional mechanisms may play a role (**Figure 3A**). A major clue supporting this possibility was given by experiments showing that Ca^2^^+^ and contractile recovery during acidosis require an intact SR and CaMKII activity, suggesting that CaMKII-dependent phosphorylation at the SR level is involved in the recovery mechanism (Pérez et al., 1995; Komukai et al., 2001; Nomura et al., 2002; DeSantiago et al., 2004; Mundiña-Weilenmann et al., 2005; Neef et al., 2013). Indeed, it was shown that phosphorylation of the Thr^17^ site of PLN transiently increased at the onset of acidosis, possibly favored by the increase in intracellular (diastolic) Ca^2^^+^ and the inhibition of phosphatase induced by acidosis (Allen and Orchard, 1983; Vittone et al., 1998). This phosphorylation was associated with the initial and most significant portion of the contractile/relaxation recovery, and both were blunted by CaMKII-inhibition (Mundiña-Weilenmann et al., 2005). Thus, CaMKII-dependent PLN phosphorylation provides a mechanism to overcome the depressant effect of acidosis on SERCA2a (Mandel et al., 1982). These increases in SR Ca^2^^+^ content and release also counteract the effect of acidosis on contractile proteins, thereby helping to maintain contractile force. More recent experiments showed that CaMKII also activates NHE which may add to the direct activation of the exchanger induced by acidosis (Vila-Petroff et al., 2010; **Figure 3A**). Interestingly, experiments by DeSantiago et al. (2004) showed absence of mechanical recovery in myocytes lacking PLN (PLNKO). This finding may be taken to indicate that PLN is essential for SR Ca^2^^+^ and mechanical recovery during acidosis. However, the effects of PLN ablation mimic maximal PLN phosphorylation. Therefore the results of DeSantiago et al. (2004) actually raise the question of whether accelerating SR Ca^2^^+^ reuptake during acidosis is beneficial, favoring mechanical recovery as discussed above, or harmful, hindering it. An explanation to these apparent contradictory results may lie on the fact that intracellular Ca^2^^+^ and mechanical recovery during acidosis require an increase in SR Ca^2^^+^ uptake above steady state, a condition that cannot be accomplished in PLNKO mice in which basal SR Ca^2^^+^ uptake is already at maximal levels. In line with DeSantiago’s results, Nomura et al. (2002) showed that the mechanical recovery from acidosis did not occur in highly phosphorylated myocytes treated with isoproterenol and a phosphatase inhibitor.

#### Acidosis and post-acidosis arrhythmias

The increase in SR Ca^2^^+^ load during acidosis, responsible for the mechanical recovery, may also increase spontaneous SR Ca^2^^+^ release and produce extra-systoles (Orchard et al., 1987). Moreover, returning to normal pH after acidosis is also arrhythmogenic: recovery of pH induces an increase in SR Ca^2^^+^ leak. This effect was attributed to the increase in the opening probability of RyR2 due to the pH increase after acidosis and the acidosis-induced increase in SR Ca^2^^+^ content, still present at the beginning of post-acidosis (Said et al., 2008). The return to normal pH also leads to recovery of the previous acidosis-induced inhibition of NCX (Philipson et al., 1982), favoring Ca^2^^+^ extrusion and Na^+^ gain into the cell, membrane depolarization and eventually triggered arrhythmias (Said et al., 2008; **Figure 3B**). Together, these results indicate that post-acidosis CaMKII-dependent DADs are triggered by two concurrent factors: (1) acidosis-induced increase in SR Ca^2^^+^ content; and (2) relief of RyR2 and NCX, previously inhibited by acidosis.

### ISCHEMIA/REPERFUSION (I/R)

#### Stunning

**FIGURE 4:**
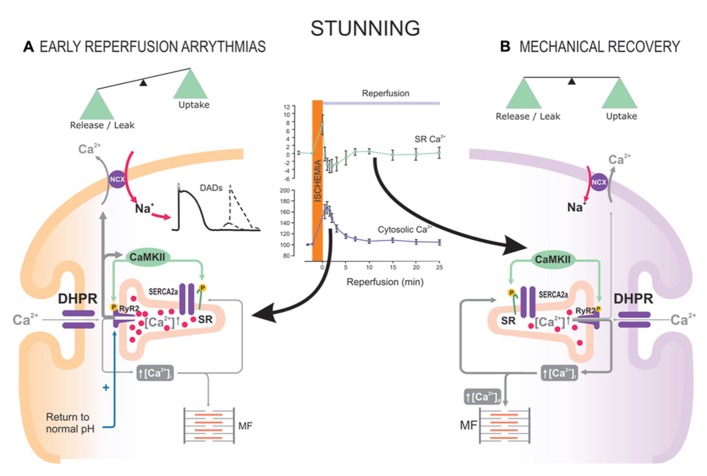
**The beneficial or detrimental effects of CaMKII activation and PLN phosphorylation in I/R depend on a tight balance between SR Ca^**2**^^+^ reuptake and leak.** Reperfusion after a short ischemic period (stunning) is associated with an increase in CaMKII-dependent PLN and RyR2 phosphorylation. During early reperfusion **(A)**, there is: abrupt release of SR Ca^2^^+^ (inset of the Figure, Valverde et al., 2010), possibly favored by the ischemia-induced increase in SR Ca^2^^+^ content; relief of RyR2 inhibition exerted by the ischemic acidosis; and increase in Ser2814 phosphorylation of RyR2 (Said et al., 2011). CaMKII-dependent phosphorylation of PLN does not counteract SR Ca^2^^+^ release, which is partially responsible for early reperfusion arrhythmias. **(B)** After the first minutes of reperfusion, the increase in Thr^17^ phosphorylation of PLN appears to successfully counteract SR Ca^2^^+^ leak, leading to Ca^2^^+^ transients and mechanical recovery.

The role of CaMKII in I/R will be addressed in detail elsewhere in this issue. We will briefly refer here to the role of CaMKII-dependent PLN phosphorylation in this pathological situation. In the last few years, a dual effect of CaMKII-dependent protein phosphorylation (beneficial and detrimental) has been described in the scenario of I/R in the intact heart. The beneficial effect of CaMKII usually refers to the intracellular Ca^2^^+^ and contractile recovery that occurs during stunning, a fully reversible post-ischemic dysfunction (Braunwald and Kloner, 1982). Initially, this beneficial effect was associated with an increase in the phosphorylation of Thr^17^ site in PLN at the onset of reperfusion (Vittone et al., 2002). Further experiments in transgenic mice in which Thr^17^ and/or Ser^16^ sites of PLN were mutated to Ala and direct measurements of intracellular Ca^2^^+^, demonstrated that Thr^17^ phosphorylation was essential for the recovery of Ca^2^^+^ transients and contractility in the stunned heart (Said et al., 2003; Valverde et al., 2006). These findings confirmed that the increase in Thr^17^ phosphorylation of PLN upon reperfusion, although transient, offers a mechanism that helps to limit cytosolic Ca^2^^+^ overload, by accelerating SR Ca^2^^+^ reuptake and thereby ameliorating intracellular Ca^2^^+^ handling (**Figure 4**). In contrast, when SR Ca^2^^+^ reuptake is highly enhanced by ablation of PLN, post-ischemic recovery of contractile function was negligible (Cross et al., 2003). A possible explanation for this apparent paradox is the higher ATP consumption of PLNKO hyperactive hearts relative to WTs, which may greatly influence contractile recovery. A second possibility that does not exclude the first one, is that under conditions in which RyR2 are altered, a persistent and exacerbated SR Ca^2^^+^ uptake, would greatly elevate SR Ca^2^^+^ content and enhance the propensity for SR Ca^2^^+^ leak, which may conspire against contractile recovery and favor reperfusion arrhythmias. Indeed, a rise in CaMKII phosphorylation of Ser2814 in RyR2 and an abrupt increase in SR Ca^2^^+^ release at the onset of reflow were recently associated with early reperfusion arrhythmias. This occurs in spite of the fact that Thr^17^ site of PLN was also phosphorylated (Said et al., 2011; Valverde et al., 2010). These results strongly suggest that the beneficial effects of increasing SR Ca^2^^+^ uptake in I/R, may turn to be deleterious under conditions in which the balance between SR Ca^2^^+^ uptake and leak is lost (**Figure 4**).

As will be discussed below, this unbalance may constitute a major cause of the detrimental effect of CaMKII activation in the irreversible I/R.

#### Irreversible ischemia/reperfusion

After a prolonged ischemic period, reperfusion evokes irreversible cardiac injury. Under these conditions, myocytes die by apoptosis, autophagy and necrosis. The rise in Ca^2^^+^ during ischemia and reperfusion leads to mitochondrial Ca^2^^+^ accumulation, which is greatly favored by the close association between mitochondria and SR and constitutes a main event in the initiation of cell death (Rizzuto and Pozzan, 2006).

Experimental evidence consistently indicates that CaMKII-inhibition is protective in the irreversible I/R injury (Zhang et al., 2005; Vila-Petroff et al., 2007; Salas et al., 2010). Although the mechanisms for myocardial protection by CaMKII inhibition are still unclear, the CaMKII deleterious pathway in I/R certainly involves the SR and the mitochondria (Vila-Petroff et al., 2007; Salas et al., 2010; Joiner et al., 2012; Valverde et al., 2013). Phosphorylation of Thr^17^, the CaMKII site of PLN, has been shown to increase at the onset of reperfusion (Vila-Petroff et al., 2007; Salas et al., 2010). This finding may suggest either that the effect of PLN phosphorylation is part of the deleterious cascade of CaMKII activation, or that this phosphorylation is beneficial, although insufficient to counteract the effect of simultaneous detrimental mechanisms. The experimental outcome of testing these possibilities has remained controversial. Referent to the first one, Yang et al. (2006) demonstrated that the protective effect of chronic CaMKII inhibition in AC3-I mice was lost, when they were interbred with PLNKO mice and submitted to myocardial infarction, supporting a detrimental effect of enhancing of SR Ca^2^^+^ uptake. Referent to the second possibility, several studies demonstrated that accelerating SR Ca^2^^+^ uptake by different means (i.e., overexpressing SERCA1a, with higher kinetics than SERCA2a, or expressing a repressor of PLN activity, PP1 inhibitor-1), alleviated post-ischemic cardiac injury (Talukder et al., 2007, 2008; Nicolaou et al., 2009), supporting a beneficial effect of accelerating SR Ca^2^^+^ uptake. A possible clue to explain these controversial findings is given by results showing that proteins, different from PLN, may be involved in the cascade by which CaMKII activity is deleterious in I/R. A decrease in the expression of RyR2 has been described in I/R (Salas et al., 2010), compatible with a degradation/damage of these channels by the concerted action of calpains and proteasomes (Pedrozo et al., 2010), that would lead to an increase in the opening probability of RyR2 (Domenech et al., 2003). Moreover, redox alterations or CaMKII-dependent phosphorylation might also influence the activity of RyR2 and SR Ca^2^^+^ leak in I/R (Hidalgo et al., 2004; Said et al., 2011; Valverde et al., 2013). These alterations would favor the unbalance between SR Ca^2^^+^uptake and leak, promoting mitochondrial Ca^2^^+^ overload and cell death. This cascade would be further stimulated by the recently described CaMKII-dependent phosphorylation of mitochondrial Ca^2^^+^ uniporter (Joiner et al., 2012). Taken together, these findings suggest that the progression toward a beneficial or detrimental effect of CaMKII activation and PLN phosphorylation in I/R would critically depend on the balance between the extent of SR Ca^2^^+^ reuptake and SR Ca^2^^+^ leak, largely given by the status/characteristics of other proteins, also involved in SR Ca^2^^+^ handling, like RyR2 (**Figure 4**).

### HEART FAILURE

Heart failure develops when the heart is unable to provide an adequate cardiac output to meet the metabolic needs of the organism. Mechanical dysfunction and arrhythmias are hallmark features of HF, being aberrant Ca^2^^+^ handling a main cause of these two characteristic alterations. Indeed, there is evidence supporting a decrease in intracellular Ca^2^^+^-transient and diminished SR Ca^2^^+^ content, an outcome that constitutes the major origin of the altered contractility in HF (O’Rourke et al., 1999; Piacentino et al., 2003), and that can be attributed to alterations in the expression/activity of different Ca^2^^+^ regulatory proteins. In particular, a decrease in SERCA2a and an increase in NCX expressions have been described in different HF models and species, including human (Hasenfuss, 1998). An enhanced SR Ca^2^^+^ leak, through hyperphosphorylated RyR2, would also contribute to the decrease in SR Ca^2^^+^ content and Ca^2^^+^ release, typical of HF (Ai et al., 2005; Shan et al., 2010; Respress et al., 2012).

The decrease in SERCA2a expression is not associated with a parallel decrease in PLN, which would produce an increase in the functional stoichiometry PLN/SERCA, with a decrease in SERCA2a Ca^2^^+^ affinity and SR Ca^2^^+^ uptake rate and a prolongation of relaxation times (Meyer et al., 1995). Moreover, phosphorylation of PLN has been found to be decreased, either at Ser^16^ (Schwinger et al., 1999; Sande et al., 2002), Thr^17^ (Netticadan et al., 2000), or both (Huang et al., 1999; Mishra et al., 2003), accounting for increased inhibition of SERCA2a. These findings may be due to the attenuation of β1-adrenergic cascade, due to receptor desensitization, down-regulation and uncoupling, typical of the disease progression (Bristow et al., 1982; Dash et al., 2001; Port and Bristow, 2001), and/or the increase in PP1 activity, described in HF (Bibb et al., 2001; Carr et al., 2002; Gupta et al., 2003). Indeed, in human failing myocardium, phosphorylation of Ser^16^ in PLN decreased because of increases in PP1 activity (Schwinger et al., 1999), whereas phosphorylation of Thr^17^ decreased due to increased activity of PP2B (calcineurin; Münch et al., 2002). Interestingly, this decrease occurred despite an increase in CaMKII activity characteristic of HF. Taken together, these results indicate that the increase in SERCA2a/PLN ratio and the diminished phosphorylation of PLN, are key determinants of the depressed SR Ca^2^^+^ uptake in HF, leading to an increase in diastolic Ca^2^^+^, a decrease in SR Ca^2^^+^ stores and therefore in Ca^2^^+^ available for contraction. This results in reduced contractile force, impaired relaxation and altered force-frequency relationship.

Given this central role of SERCA2a and PLN in the defective Ca^2^^+^ handling typical of HF, the strategy of altering SERCA2a and/or PLN levels or activity to restore perturbed Ca^2^^+^ uptake into the SR are potential therapeutic strategies for HF treatment (del Monte and Hajjar, 2003). Indeed, overexpression of SERCA2a can restore Ca^2^^+^ handling and contractile function in animal models (Cutler et al., 2012) and in human HF (del Monte et al., 1999; Jaski et al., 2009), suggesting that repairing SERCA2a expression may be a viable therapy. Moreover PLN ablation prevented HF in a mouse model of dilated cardiomyopathy caused by deficiency of the muscle-specific LIM protein (Arber et al., 1997; Minamisawa et al., 1999). In isolated human HF myocytes, gene therapy with antisense against PLN improved contractile and diastolic function (del Monte et al., 2002). In contrast, PLN ablation increased SR Ca^2^^+^ filling and contractility in mice with cardiomyopathy attributable to overexpression of CaMKII. This led to premature death and mitochondrial Ca^2^^+^ overload, suggesting that accelerating SR Ca^2^^+^ uptake and increasing SR Ca^2^^+^ load, is disadvantageous at least in the presence of excessive CaMKII activity (Zhang et al., 2010). These findings are consistent with the idea already discussed for I/R: in the face of phosphorylated RyR2 channels, as is the case of CaMKII overexpressing mice, repletion of Ca^2^^+^ stores through PLN ablation could further worsen overall heart function, via mitochondrial Ca^2^^+^ loading, cell death, and arrhythmias.

## CONCLUDING REMARKS

We have described that PLN and its CaMKII-dependent phosphorylation are part of a multimeric functionally coupled signaling complex, composed of SERCA, PLN, HAX-1, PKA, CaMKII, PP1, I-1, and Hsp20, which reversibly regulates SR Ca^2^^+^ cycling. Although CaMKII-dependent PLN phosphorylation contributes to β1-adrenergic mechanical response, its role is subordinate to the PKA-dependent increase in cytosolic Ca^2^^+^ and inhibition of phosphatase, necessary to activate CaMKII and phosphorylate Thr^17^ of PLN. These requirements are also achieved under different pathological situations, like acidosis and I/R, independent of PKA activation. Under these conditions, CaMKII-dependent PLN phosphorylation may paradoxically produce either favorable or harmful cardiac effects. The findings summarized in this review also suggest that the beneficial or detrimental effects associated with CaMKII activation and PLN phosphorylation depend on a tight balance between SR Ca^2^^+^ reuptake and leak, determined by the status/characteristics of other SR proteins, among which the RyR2 is a main candidate. A moderate or even high increase in SR Ca^2^^+^ uptake (and content) due to PLN phosphorylation, would enhance RyR2 opening due to the regulatory effect of intra-SR Ca^2^^+^. However, in the absence of additional RyR2 modifications, the increased SERCA2a activity, produced by PLN phosphorylation, may cope with the enhanced diastolic SR Ca^2^^+^ release/leak. In contrast, even moderate increases in SR Ca^2^^+^ may increase diastolic SR Ca^2^^+^ release under conditions where RyR2 activity is altered independently of intra-SR Ca^2^^+^-induced modifications, enhancing the propensity to arrhythmias and leading to mitochondrial Ca^2^^+^ overload, which favors apoptosis and necrosis. Thus, increasing SERCA2a activity by PLN phosphorylation seems to have the potential of producing salutary effects in a number of diseases, as long as these effects are achieved under conditions in which diastolic Ca^2^^+^ release is satisfactorily controlled. Future research in this area is needed to parse the contribution of different players involved in the balance/interaction between SR Ca^2^^+^ reuptake and leak, including the RyR2 regulators or the L-type Ca^2^^+^ channels. More specifically to the subject of this review, the recently described multimeric SERCA/PLN-ensemble may represent a nodal point in the interaction of several protein partners, regulating and modifying the fine-tuned control of Ca^2^^+^ cycling achieved by the duo SERCA-PLN. An intensive scrutiny of the various proteins of this new pathway will give new insights into their role in SR Ca^2^^+^ uptake control and may provide novel therapeutic avenues which can contribute to solve the abnormalities in Ca^2^^+^ handling underlying different pathological process.

## Conflict of Interest Statement

The authors declare that the research was conducted in the absence of any commercial or financial relationships that could be construed as a potential conflict of interest.
